# Neuroenergetics of traumatic brain injury

**DOI:** 10.2217/cnc.15.9

**Published:** 2015-09-22

**Authors:** Kate Karelina, Zachary M Weil

**Affiliations:** 1 Department of Neuroscience, The Ohio State University Wexner Medical Center, Columbus OH, USA

**Keywords:** central metabolism, insulin resistance, neurodegeneration, neuroinflammation, TBI

## Abstract

A subset of traumatic brain injury (TBI) patients exhibit cognitive deficits later in life which may be due to the underlying pathology associated with Alzheimer's disease (AD) or chronic traumatic encephalopathy. The similarities between chronic traumatic encephalopathy and AD merit investigation of potentially similar mechanisms underlying the two diseases. Experimental and clinical studies of AD brains have revealed that insulin resistance links metabolic dysfunction to the neurodegeneration and cognitive deficits associated with AD. Recent work in experimental TBI has established that recovery is dependent on the return of normal brain metabolism and mounting evidence for a role of brain insulin in regulating central metabolism suggests that TBI, like AD, results in central insulin resistance. Here, we review the converging evidence from AD, TBI and diabetes research linking insulin insensitivity to neurodegeneration.

## Traumatic brain injury

Traumatic brain injuries (TBIs) are increasingly recognized as an important global health concern and represent the leading cause of disability and death worldwide [1]. In the USA alone, there are, on average, 2.5 million documented head injuries annually, although the true number probably far exceeds that value [2]. The estimated economic costs of TBI (in 2000 dollars) approaches US$60 billion including healthcare costs and lost productivity [3]. At least 5.3 million Americans (nearly 2% of the total population) are living with long-term disability associated with TBI [4]. In addition, there is mounting evidence that individual, and in particular repeated, head injuries greatly increase the possibility of other diseases and disabilities, further increasing the overall societal costs [5]. These epidemiological data indicate the importance of both TBI prevention and the development of treatment and rehabilitation strategies for this population.

By definition, TBI involves an external mechanical force that causes brain dysfunction. Broadly, TBI can be divided into two categories, focal injuries due to contusions, lacerations, penetrating ballistic objects or intracranial hemorrhage and diffuse injuries that typically occur after impact acceleration or explosive blasts [6]. The biomechanical forces associated with these two types of injuries are obviously very different. Furthermore, head injuries are extremely heterogeneous and outcomes can vary dramatically based on the severity of impact, age, comorbidities and genetics among other variables. Therefore, TBI is really a broad diagnosis that includes a large number of mechanical head injuries; however, all CNS trauma outcomes are determined by both primary and secondary phases of damage. Primary injuries are the direct result of mechanical damage to the CNS, whereas secondary injury represents the pathological responses to the initial injury that can greatly exacerbate tissue damage and long-term deficits [7].

TBI rapidly disrupts neuronal homeostasis. The mechanical deformation of brain tissue can result in membrane shearing, neuronal death, rapid and spreading depolarization, increases in excitatory amino acid release and axonal disconnections [8,9]. In the first few minutes after TBI, neuronal survival is dependent on a rapid restoration of membrane potentials and ionic homeostasis. If cells cannot rapidly repolarize their membranes, the resulting ionic imbalances can lead to swelling and lysis of neurons, neurites and glia [7].

Thus, control of inflammatory responses is critical to TBI outcomes but is energetically expensive. This crisis situation is often exacerbated by hemorrhages, impairments in cerebral perfusion and autoregulation, and diminished neuronal capacity to utilize energy. Over time after the initial injury, metabolic dysfunction and inflammatory responses mutually reinforce one another. The goals of this article are to review the literature on the role of central metabolic dysfunction in TBIs and further discuss how this process affects long-term outcomes following TBI.

## Cerebral metabolism after TBI

The pathophysiology of TBI is complex, but involves diffuse axonal injury, frank neuronal death, inflammation, and persistent metabolic abnormalities. There is a consistent phenomenon across brain injury subtypes that the capacity for the brain to utilize energy (namely glucose) is significantly modulated following injury. Most studies have reported a transient period of hypermetabolism followed by a prolonged hypometabolic phenotype [10–12]. This phenomenon has been reported in both animal and human studies. The initial hypermetabolism is apparently in response to the release of excitatory amino acids immediately following injury and may be necessary to recover some aspects of homeostasis in the injured brain [10]. In an effort to investigate the role of metabolic dysfunction as a mediator of enhanced vulnerability to repeated injury after TBI, we recently identified that a single impact (weight drop) led to a significant increase in brain glucose utilization in mice by postinjury day 6, and by 10 days brain glucose utilization dropped below baseline and remained low through the latest time point examined (20 days). On the other hand, in mice injured twice, 3 days apart, no significant change in brain glucose utilization was observed at any time point examined, indicating an inability of the brain to meet increased metabolic demands. Finally, if the two injuries occurred further apart (20 days apart), the pattern of increased glucose utilization after the second impact paralleled the increase that occurred after just one impact. These data suggest that a longer recovery period between impacts corresponds to an enhanced ability of the brain to mount the appropriate metabolic response to the second injury. This pattern of glucose utilization was associated with cognitive function after injury, with the group injured twice (3 days apart) exhibiting the greatest learning/memory deficits, while the other groups recovered cognitive function over time [13]. Thus, the acute period following TBI can be conceptualized as a metabolic crisis, wherein the energetic demands on the injured brain are increased to allow for rapid recovery. In severe or repeated TBI, there is simultaneously the need to maintain cellular integrity in the face of excess glutamate release and mechanical injury to the cell membranes, and a reduced capacity to utilize energetic substrates, resulting in a reduced capacity for tissue repair and functional recovery. The recovery of cognitive and executive function correlates strongly with the return of normal glucose metabolism in both humans and animals [13–15], however, few studies have definitively linked alterations in glucose metabolism to functional outcome following TBI.

Until relatively recently, energy metabolism inside the CNS was not believed to be regulated in an insulin-dependent manner, and in fact CNS cells were thought to be devoid of insulin all together. It is now well understood that insulin is both transported into the CNS across the blood–brain barrier and synthesized locally [16–18]. Moreover, brain insulin receptors are densely distributed throughout the brain, including the olfactory bulbs, cerebral cortex, hippocampus, hypothalamus, amygdala and septum [19]. Insulin receptor signaling in the CNS differs from that in peripheral tissues in that insulin does not directly upregulate glucose transporter gene expression in neurons and thus does not directly increase neuronal glucose uptake. Rather, insulin modulates neuronal physiology via MAPK and PI3K signaling [20], which in turn alter aspects of metabolism and promote neuronal regeneration and survival via phosphorylation of Akt. Insulin receptor sensitivity is regulated in two general ways. First, ligand-dependent receptor activation induces phosphorylation of the IRS-1 protein [21]. Phosphorylation at specific epitopes reduces the interaction between IRS-1 and the membrane-bound insulin receptor and prevents further signaling [22]. This is a homeostatic negative feedback system to regulate cellular responses to insulin [23].

**Figure 1.**
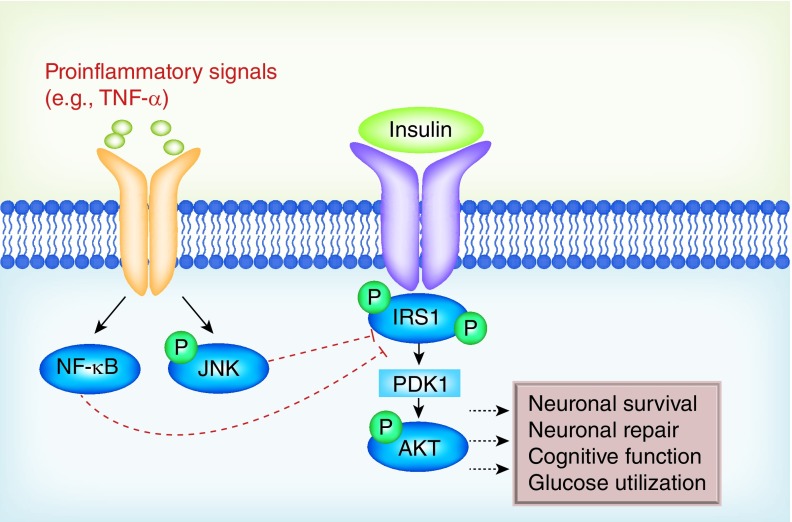
**Neuroinflammation and insulin resistance in the CNS.** Inflammatory events, such as those induced by traumatic brain injury, activate signaling cascades (including NF-κB and JNK) that phosphorylate and inactivate insulin receptor substrate proteins. Inactivation of insulin receptor substrate proteins, in turn, prevents insulin receptor signaling. One prominent downstream target of insulin signaling is AKT, a neuroprotective kinase that also influences neuronal energy metabolism. Insulin resistance following traumatic brain injury therefore deprives the nervous system of this protective pathway, rendering the individual vulnerable to neurodegeneration and cognitive deficits.

In addition, a huge variety of inflammation-, trauma- and danger-associated signals including cytokines, chemokines, tau and heat shock proteins can also lead to the phosphorylation of IRS-1 in a ligand-independent manner [24,25]. In particular, the proinflammatory cytokine TNF-α provides a clear mechanistic link between acute trauma-induced neuroinflammation and metabolic dysfunction [26–29]. A primary (though not exclusive) regulator of TNF-α-induced inflammatory responses is the transcription factor NF-κB, which has been shown to be involved in the etiology of insulin resistance and Type 2 diabetes [30]. TNF-α and other inflammatory processes also promote IRS-1 phosphorylation via activation of the JNK pathway [31]; for in-depth reviews, see [28,32,33]. In addition to the direct effects of neuroinflammation, TBI also produces widespread axon degeneration [34]. Both insulin and IGF-1 are known to play a significant role in the regulation of myelin synthesis [35,36], and treatment with IGF-1 has been shown to protect oligodendrocytes in animal models of excitotoxicity and cerebral hypoxia-ischemia [37,38]. Taken together, the relationship between TBI-induced neuroinflammation and the resulting pathophysiology/cognitive deficits are likely mediated in part by the direct disruption of central metabolic processes (Figure 1).

Dysregulation of metabolic processes is detrimental to TBI recovery [39]. Hyperglycemia at the time of hospital admission is associated with poor outcome and greater mortality rates in TBI patients [40,41]. Not surprisingly, recent clinical reports reveal that obese TBI patients (who commonly present with insulin resistance in the form of Type 2 diabetes) experience more complications (i.e., multiple organ system failure, acute respiratory distress syndrome, myocardial infarction and deep vein thrombosis) and require longer hospital stays and more medical interventions (i.e., mechanical ventilation, dialysis) compared with nondiabetic controls [42]. Diabetes is also a significant predictor for mortality after TBI [43]. Several clinical trials targeting glucose control in TBI patients have been conducted. Early results indicate some improvement with intensive insulin therapy at the time of hospitalization: some studies link insulin treatment to reduced infection rates and shorter hospitalizations, however, results have been mixed and mortality rate, particularly among severe TBI patients, does not improve with intensive insulin therapy [44–46].

Nonetheless, in animal models, insulin is well understood to be a potent neuroprotectant that can promote neuronal survival and recovery following a variety of insults to the CNS [47,48]. In rats, treatment with insulin or IGF-1 attenuates ischemic brain damage [49] in addition to improving motor and cognitive recovery following TBI [50]. Such patterns of recovery can be attributed in part to the role of IGF-1 as a regulator of neuronal growth and differentiation, as well as its ability to promote synaptic plasticity and neurogenesis [51,52]. This makes insulin a highly attractive treatment option for brain injury, unfortunately although insulin is neuroprotective both *in vivo* and *in vitro*, it is limited as a therapeutic target for two key reasons. First, systemic insulin receptor activation stimulates glucose uptake by the liver and muscle tissues and can therefore induce hypoglycemia, which is itself very damaging to the compromised brain. Second, insulin therapy is significantly limited by the phenomenon of insulin resistance that is induced by both acute and chronic disease processes. Therefore, significant efforts need to be put toward developing treatments that attenuate injury-induced insulin resistance, leading to enhanced insulin receptor sensitivity. Moreover, the role of insulin signaling in mediating cognitive deficits after TBI remains unknown, thus a more complete understanding of these phenomena will be necessary in order to establish a treatment plan targeting central metabolic processing in TBI patients.

## Cognitive decline after TBI

Repeated TBI is closely linked both pathophysiologically and epidemiologically to chronic neurodegenerative disease. For instance, cognitive disabilities following TBI vary largely based on severity, age and general health of the individual, and the degree of cognitive impairment following a single TBI ranges from mild temporary cognitive impairment to Alzheimer's-like cognitive deterioration. Both the number of lifetime TBIs and their severity predict cognitive impairment, particularly learning and memory. In the majority of cases, patients that have experienced a single mild TBI recover to their baseline cognitive performance within a few months, whereas patients with moderate or severe TBI are more likely to exhibit neurological symptoms years later [53]. However, some patients (particularly those with the APOE-ε4 allele) are at an increased risk of developing AD after a single mild TBI, and the risk increases substantially in individuals with a history of severe TBI [54–56].

In terms of cognitive symptoms, many TBI patients struggle with disturbances of attention, memory storage and retrieval, planning and organization, social interaction and motivation [57]. Often, trauma-related functional deficits can be linked to brain regions directly affected by a penetrating or focal brain injury [58,59], however, the development of cognitive impairments following nonpenetrative mild injuries implicates the involvement of a broad underlying pathophysiology. Indeed, great effort has been put into identifying the role of various TBI-induced cytotoxic processes (i.e., neuroinflammation, oxidative stress, excitotoxicity) and direct neuronal injury (axonal degeneration) in mediating post-trauma cognitive impairments [8,60–62]. Unfortunately, treatments that target these processes have not resulted in consistent cognitive recovery after TBI. Coupled with reports of increasing TBI rates in both male and female athletes, members of the armed forces and other civilians, there is now a greater need than ever to identify the mechanisms underlying trauma-induced cognitive decline.

Individuals that have experienced repetitive concussive or subconcussive brain injury are at an increased risk of developing a neurodegenerative disorder called chronic traumatic encephalopathy (CTE), which closely resembles AD [63]. Acute symptoms of CTE manifest as confusion, mild memory loss, reduced concentration and attention as well as dizziness and headaches. Over time these symptoms progress to the point of overt dementia, including lack of insight and poor judgment, language difficulty, aggression and irritability [64]. This cluster of symptoms had been historically described in boxers as ‘punch drunk’ [65] or ‘dementia pugilistica’ [66], and is today recognized as a serious consequence of repeated brain trauma in athletes, members of the military and law enforcement and even victims of physical abuse [67–69]. The presentation of CTE is distinct from other trauma-related symptoms of cognitive decline (i.e., postconcussion syndrome) in that, like AD, CTE is a neurodegenerative disease. Characteristic pathological features of CTE include tau-positive neurofibrillary tangles along superficial frontal and temporal cortices, in sulci, and along the neurovasculature, as well as an accumulation of tau-immunoreactive astrocytes [70–73]. Diffuse deposition of amyloid-β plaques, a hallmark of AD, occurs in fewer than half of CTE cases [70]. Although the specific manifestations of AD and CTE neuropathology are distinct enough to be distinguishable from one another, the striking similarity of symptoms merit careful investigation of a potential common underlying cause for both.

## Central insulin resistance in neurodegenerative disease

Currently, relatively little is known about brain insulin signaling following TBI, particularly as it relates to neurodegeneration and cognitive decline. However, insulin is a highly attractive therapeutic target in neurodegenerative disease, given its role in improving memory [74–76], neuroprotection [47–49] and neurogenesis [77], as well as its anti-inflammatory properties [78]. An emerging body of evidence is now beginning to link both the pathophysiology and clinical symptoms of AD to insulin resistance. Early clinical studies identified peripheral hyperinsulinemia and poor glucose regulation in AD patients [79,80]. This led to large-scale studies that established Type 2 diabetes as a significant risk factor for the development of AD [81,82]. Parallel to these studies were a series of investigations into altered cerebral glucose metabolism in AD. PET scans indicate a significant parietotemporal hypometabolism in patients with AD dementia [83,84]. The impairments in cerebral glucose metabolism occur early in AD, often preceding initial symptoms and deteriorate further with the progression of AD [85]. Postmortem analysis of brain tissue from AD patients also indicated a progressive reduction of brain insulin receptor and IGF receptor mRNA, both of which showed up to an 85% reduction in late stage AD. Receptor binding assays confirmed reduced insulin and IGF binding commensurate with AD severity [86]. Additional alterations to the insulin signaling pathway in AD patients involve changes in the distribution patterns and morphology of insulin receptors [87], IGF-1 receptor insensitivity, and basal elevation in IRS-1 phosphorylation [88].

Converging evidence from animal models has greatly furthered our understanding of the role that insulin signaling plays in cognition and neurodegenerative disease. Insulin treatment is well known to improve learning and memory through mechanisms involving signaling cascades downstream of the insulin receptor [75,89] in both rodents and humans [74,76,90]. Conversely, experimental inhibition of peripheral insulin signaling (a model of diabetes) impairs memory [91,92], while treatments that enhance insulin sensitivity improve memory deficits [93,94]. Moreover, experiments using mouse models of Alzheimer's disease report that insulin resistance promotes amyloidosis [95,96], likewise amyloid-β production has been shown to promote insulin resistance [97,98]. Finally, transgenic animals that lack brain IRS-2 [99], insulin [100] or neuron-specific insulin receptors [101] display hyperphosphorylation of tau and neurofilament, increased cell death, and cognitive deficits.

Taken together, the epidemiological and clinical research are uncovering concrete evidence of a relationship between AD and insulin resistance [102,103]. Indeed, the relationship between insulin signaling and AD may turn out to be not just a therapeutic target but also an early detection marker for AD [104]. Moreover, the recent surge of interest in central insulin signaling has identified a role for insulin dysfunction in the pathophysiology of additional neurodegenerative diseases, including vascular dementia [105,106], Parkinson's [107] and Huntington's diseases [108], thus potentially uncovering a novel therapeutic approach. As mentioned above, direct insulin treatment is undesirable for this group of disease states given the potential for creating a state of hypoglycemia and reduced efficacy as a function of insulin resistance associated with neurodegenerative disease. However, a few clinical trials using intranasal insulin administration have reported successful cognitive outcomes in AD or mild cognitive impairment patients [109–111]. However, pharmacological approaches aimed at enhancing insulin receptor sensitivity have seen success in neurodegenerative diseases. One such class of drugs includes PPAR-γ agonists, which significantly improve insulin sensitivity by increasing the production of glucoregulatory proteins [112]. Selective PPAR-γ agonists, such as rosiglitazone and pioglitazone, improve cognitive performance and reduce amyloid-β in patients with mild AD [113,114], and produce promising behavioral and neuroprotective effects in animal models of Parkinson's disease [115], amyotrophic lateral sclerosis [116] and Huntington's disease [117]. Follow-up clinical trials using PPAR-γ agonists in mild-to-moderate AD have had minimal success owing in part to variability in the progression of AD and small sample sizes. Thus, although these drugs are generally well tolerated and have a good safety profile, their use is not widely recommended for the treatment of AD [118–120]. Similar pharmacologic approaches have recently been successfully implemented in animal models of TBI. For example, the incretin GLP-1 is a peptide that controls blood glucose [121]. Through its action on the GLP-1 receptor (GLP-1R), GLP-1 promotes pancreatic β-cell proliferation, inhibits β-cell apoptosis and increases insulin secretion [121,122]. Importantly, GLP-1Rs are expressed throughout the brain and central GLP-1 can both control whole-body insulin sensitivity [123] and promote neuroprotection [124]. Heile *et al.* [125] transplanted GLP-1 transfected mesenchymal stem cells into the lateral ventricles of rats prior to TBI. The encapsulated stem cells produced GLP-1 throughout the duration of the experiment, and significantly reduced cell death and neuroinflammation [125]. Incretin mimetics represent a promising treatment strategy for AD and have been shown to improve neuronal plasticity through mechanisms involving normalization of neuronal metabolic activity [126,127]. The GLP-1R agonist, exendin-4, inhibits the development of insulin resistance in a mouse model of AD [128]. Exendin-4 was also recently used in a mouse model of mild TBI: whether administered prior to or following the TBI, treated animals exhibited improved visual and spatial memory relative to controls [129–131]. A clinical trial on the effects of liraglutide (a GLP-1 receptor agonist) on cognition and neurodegeneration in AD patients is currently underway [132]. Taken together, these studies add to the growing and compelling evidence of the contribution that insulin sensitivity may have in mediating both TBI-induced pathophysiology and the resulting cognitive deficits.

TBI in both clinical and experimental populations is associated with a temporary but significant derangement of metabolic function. This impairment in brain energy metabolism appears to correlate temporally with both enhanced vulnerability to repeated injury and recovery from the initial insult. Given the relationship between AD and TBI, it seems plausible to propose that mechanical injuries to the nervous system induce long-lasting events in the brain that recapitulate some of the pathophysiology of AD neurodegeneration. These events, including inflammation, impairment in the utilization of metabolic fuels and the development of tau deposits, bear striking similarity to the neurodegenerative process that results in AD. Therefore, understanding the pathophysiology that links TBI to AD or CTE, with a particular focus on energy metabolism, is likely to have significant benefits for both conditions as well as potentially providing biomarkers to guide clinicians in return-to-work/play decisions in TBI.

## Conclusion & future perspective

Despite significant progress in identifying the signs of and treating overt symptoms of TBI, clinicians and researchers continue to struggle with the long-term consequences of repeated and severe brain injuries. The neuropathology and associated cognitive deficits following TBI are well described as they relate to injury-induced neuroinflammation, however the development of neurodegenerative disease years after the neuroinflammation has resolved represents a gap in our understanding of the mechanisms by which this occurs. Long-lasting changes in brain metabolism are now understood to play a significant role in mediating the brain's ability to recover and regenerate damaged tissue after TBI. A greater understanding of these mechanisms may allow both intervention for the prevention of long-term neurodegeneration, and development of biomarker assays to delineate severity and further inform treatment and rehabilitation strategies.

**Executive summary**

- Traumatic brain injuries cause dysregulation of brain glucose metabolism and can result in the development of the neurodegenerative disease chronic traumatic encephalopathy.
- Similar neurodegenerative diseases, such as Alzheimer's disease, are associated with central metabolic dysfunctions mediated by the development of brain insulin resistance.
- Pharmacological reinstatement of brain insulin sensitivity can rescue both neuronal damage and cognitive deficits in rodent models of experimental traumatic brain injury.
